# Human Bocavirus Infection of Permanent Cells Differentiated to Air-Liquid Interface Cultures Activates Transcription of Pathways Involved in Tumorigenesis

**DOI:** 10.3390/cancers10110410

**Published:** 2018-10-30

**Authors:** Verena Schildgen, Monika Pieper, Soumaya Khalfaoui, Wolfgang H. Arnold, Oliver Schildgen

**Affiliations:** 1 Kliniken der Stadt Köln gGmbH, Institut für Pathologie, Kliniken der Privaten Universität Witten/Herdecke mit Sitz in Köln, Ostmerheimer Str. 200, D-51109 Köln/Cologne, Germany; pieperm@kliniken-koeln.de (M.P.); khalfouis@kliniken-koeln.de (S.K.); 2 Universität Witten/Herdecke, Lehrstuhl für Biologische und Materialkundliche Grundlagen der Zahnmedizin, D-58448 Witten, Germany; wolfgang.arnold@uni-wh.de

**Keywords:** human bocavirus, transcriptome analyses, tumorigenesis

## Abstract

The parvoviral human bocavirus (HBoV) is a respiratory pathogen, able to persist in infected cells. The viral DNA has been identified in colorectal and lung tumors and thus it was postulated that the virus could be associated with tumorigenesis. This assumption was supported by the fact that in HBoV-infected patients and in an in vitro cell culture system, pro-cancerogenic and -fibrotic cytokines were expressed. In this work, it is shown by a whole transcriptome analysis that, also at the mRNA level, several pathways leading to neoplasia and tumorigenesis are significantly upregulated. In total, a set of 54 transcripts are specifically regulated by HBoV, of which the majority affects canonical pathways that may lead to tumor development if they become deregulated. Moreover, pathways leading to necrosis, apoptosis and cell death are downregulated, supporting the hypothesis that HBoV might contribute to the development of some kinds of cancer.

## 1. Introduction

Cancer is one of the leading causes of death worldwide. Meanwhile, several risk factors, such as environmental pollution, unhealthy lifestyle, inherited gene mutations, and chronic infections, have been identified, but for many cancer entities, the underlying processes of tumor development are not yet sufficiently understood. In this context, bocaviral DNA was detected in about 20% of colorectal and lung cancers [1], a finding that was meanwhile confirmed several times [2,3,4]. The human bocavirus (HBoV) is the second human pathogen parvovirus, which is related to the bovine parvovirus and the minute virus of canine (MVC). It is mainly associated with respiratory infections in all age groups (subtype 1) (reviewed by [5,6]), but may also display gastrointestinal symptoms (subtypes 2–4) (reviewed by [5]). After the identification of a covalently closed circular DNA [7] that most likely represents the form of a persisting genome, we and others have shown that HBoV is able to persist and induce long lasting infections in adults that result in a subclinical asymptomatic course [8,9,10,11] or that may go ahead with chronic symptoms like cough [12]. These data were confirmed by several findings from other groups [13,14,15] and led to the hypothesis that the virus may take a related pathophysiological route like the human hepatitis B virus by inducing fibrosis, followed by cancerogenesis in its target organs [16].

Possible pathological mechanisms could be, on the one hand, a direct involvement in tumorigenesis by triggering cancerogenic pathways through hit-and-run mechanisms or, on the other hand, an indirect influence due to a chronic subclinical infection. Based on this assumption, a previous analysis of cytokine profiles in bronchoalveolar lavages (BALs) of HBoV-infected patients showed the expression of profibrotic and procancerogenic cytokines and chemokines, a result which was consistent with observations in CuFi-8 cell culture experiments [17]. Due to these findings, we identified pathways deregulated by HBoV infection, which are associated with the development of fibrosis and cancerogenesis. Therefore, we used the established CuFi-8 cell culture model [17,18,19,20,21] and analyzed transcription alterations, specifically induced by the HBoV infection, by whole transcriptome analyses with next-generation sequencing. 

## 2. Results

**Figure 1:**
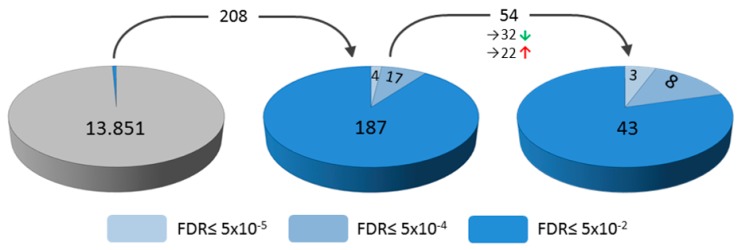
Analyses of transcriptome alterations in CuFi cells. Alterations during human bocavirus (HBoV) infection of mock-infected CuFi-8 cells vs. HBoV-infected CuFi-8 cells. A total number of 208 genes out of 13,851 genes were regulated with statistically approved significance (False Discovery rate FDR ≤ 0.05). Of these 54 genes were HBoV-specific regulated. These 54 genes have been identified by comparison of mock-infected CuFi-1 and -5 transcriptomes with the mock-infected CuFi-8 transcriptome. Therefore, transcripts of HBoV-infected CuFi-8 cells, of which the RNA level in mock-infected CuFi-8 differs from mock-infected CuFi-1 and CuFi-5 cells, were excluded from the set of 208 transcripts. Of the 54 genes, 22 were upregulated, while 32 were downregulated. The experiments were separately performed in triplicate, based on three independent infections per cell line.

The aim of the study was to identify signaling pathways that are triggered by the HBoV infection. Therefore, CuFi-8 cells were infected with HBoV or mock transfected as controls. Cells were harvested and subjected to RNA extraction five days after infection. The RNA was used for a whole transcriptome analysis based on next-generation sequencing analyses. A total number of 13,851 transcripts were identified, of which 208 were statistically significant regulated in HBoV-infected CuFi-8 cells (False Discovery rate FDR ≤ 0.05), compared to the mock infections (Figure 1). 

**Figure 2:**
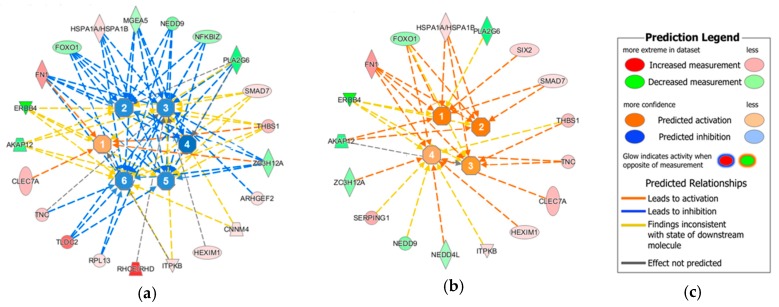
Core analyses network predictions for HBoV-infected CuFi-8 cells. This analysis shows that the 54 HBoV-specific genes are involved in apoptosis, necrosis and (re-)organization of the extracellular matrix. (**a**) Interaction of the different genes with phosphorylation processes (1), apoptosis (2), cell death in general (3) and of pancreatic cancer cells (4) and tumor cell lines (5) in particular, as well as necrosis (6). (**b**) Influence of the HBoV-specific genes on the organization of connective tissues, including growth (1), proliferation (2) amongst others of fibroblasts (3), and the quantity of cells (4). (**c**) Prediction legend. Orange indicates an upregulation, and blue represents a downregulation of the respective pathway. Grey indicates that no pathway alterations based on single transcripts could be predicted. Brighter color indicates a weaker alteration, whereas darker color indicates a strong regulation.

Among these genes were *ERBB4*, *FOXO1*, and *AKAP12* as downregulated transcripts, whereas *HSPA1A/1B*, *FN1*, *TNC*, and *HEXIM1* were upregulated (Figure 2). All of them are associated with neoplasia, tumorigenesis, fibrosis, as well as apoptosis if their regulation differs from normal physiological conditions.

In order to exclude any cell type- or host-specific effects, RNA was also isolated from mock-infected CuFi-1 and CuFi-5 cells. These cells are highly similar to CuFi-8 cells, but originate from different donors and do not productively support the replication of HBoV. All CuFi cells were immortalized by dual retroviral infection with HPV-16E6/E7-LXSN and hTERT-LXSN. In order to exclude general effects by this procedure, we decided to subtract the background and to focus on mechanisms that are not donor-specific. In CuFi-1 and CuFi-5, 1601 out of 14,861 transcripts were regulated with statistically approved significance compared to HBoV-negative CuFi-8 cells, of which 800 were downregulated and 801 were upregulated. Seven transcripts were only identified in CuFi-8 cells: membrane protein hyaluronidase 4 (*HYAL4*), *LINC00920* (non-coding RNA), *LOC102723568* and *LOC102723342* (two RNAs of unknown function), the transcription factor *POU2F2*, the blood group antigen *RHCE*, and the intracellular protein *TLDC2*, which is related to the nuclear receptor family.

After these initial analyses, the transcriptomes of CuFi-1 and CuFi-5 were compared with the transcriptomes of mock- and HBoV-infected CuFi-8 cells. Out of the 208 transcripts regulated during HBoV infection in CuFi-8 cells, only 54 genes were equally expressed in all three non-infected CuFi cell lines (Figure 1). Consequently, the expression of this remaining set of 54 genes (Table 1) has to be regulated specifically by the HBoV infection in CuFi-8 cells.

In order to identify any regulated intracellular networks influenced by the HBoV infection, an extended bioinformatics analysis was performed using the Ingenuity Pathway Analysis (IPA) platform, an established internet-based analysis tool for the analysis, interpretation and mechanistic prediction of NGS-datasets [22].

The overall analysis of regulated transcripts reveals that the major regulated disease networks predicted by the IPA software include upregulation in these pathways that may push the HBoV-infected cell towards fibrosis and precancerous conditions (Figure 2b), while apoptotic and necrotic pathways are downregulated or inhibited (Figure 2a). The pathways, leading to increased phosphorylation of proteins, are also upregulated, as well as pathways leading to cell proliferation. For example, the increased expression of *FN1*, as well as the decreased expression of *FOXO1*, on the one hand, leads to a downregulation of apoptotic mechanisms, and on the other hand, to an increased proliferation (Figure 2).

**Figure 3:**
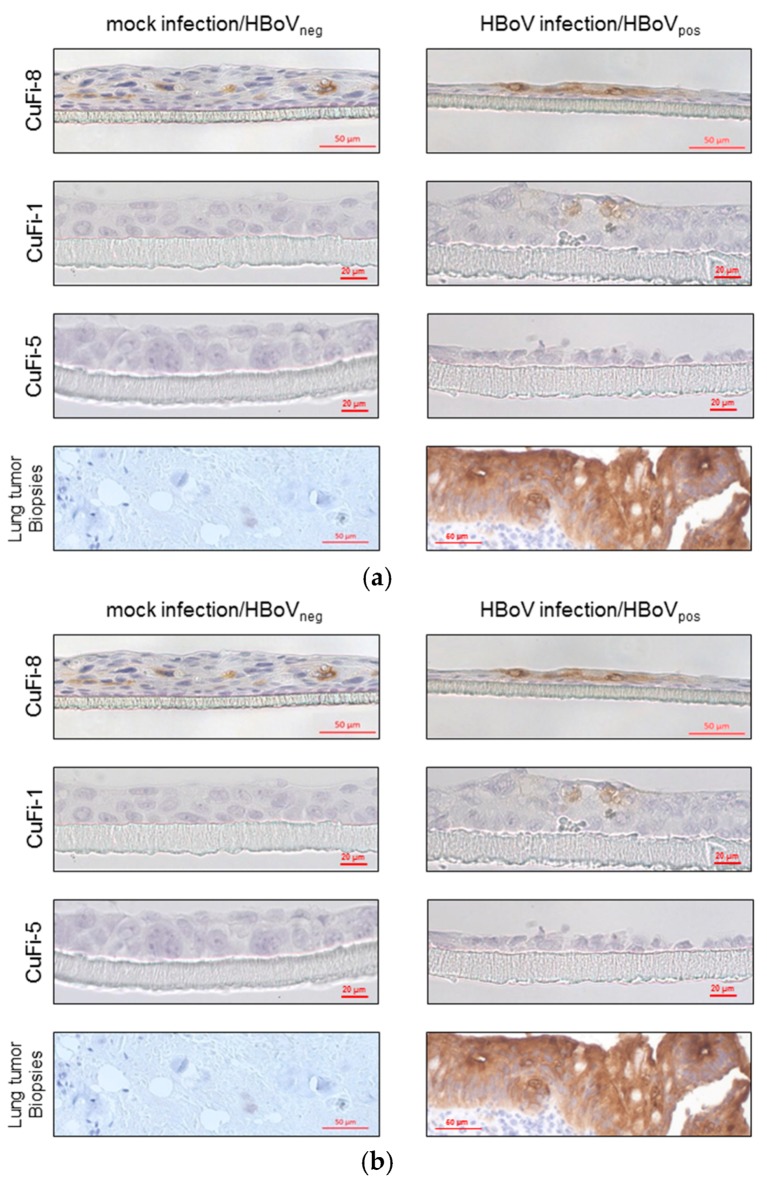
Immunohistochemical staining of CuFi-8 air–liquid-interface cultures. (**a**) Staining of CEA was prominent in HBoV-infected CuFi-8 cells and HBoV-positive lung tumor biopsies, whereas mock-infected CuFi-8 cultures, as well as HBoV-negative lung tumors, were CEA-negative. CuFi-1 and Cufi-5 cells were not CEA-positive at all. (**b**) PAS–Alcian blue staining reveals higher production of acid mucins in CuFi-8 cells compared to those in CuFi-1 and CuFi-5 cells in general. Beyond that, there is an increased expression of acid mucins after HBoV infection in CuFi-8 cells.

Besides, *FN1*, other transcripts like *Tenascin C* (*TNC*), and *THBS1* involved in the extracellular matrix (ECM) metabolism have been identified as HBoV specifically regulated. In this context, we also analyzed immunohistochemically the expression of *CEA* in HBoV-positive tumors and cell cultures compared to HBoV-negative samples and observed that the *CEA* staining was intensive in HBoV-infected CuFi-8 cells and HBoV-positive lung tumor biopsies, whereas mock-infected CuFi-8 cultures as well as HBoV-negative lung tumors are *CEA*-negative (Figure 3).

In order to identify molecular pathways, which may affect the progression to subsequent diseases due to the HBoV infection, we analyzed to what extend canonical pathways are affected by HBoV-dependent alterations of the expression profile. Accordingly, we refer to these disease pattern with a statistically significant *p*-value (*p* ≤ 0,01) predicted by the IPA core analyses that include 9 to 43 regulated transcripts, respectively, out of the 54 identified transcripts specifically regulated by the HBoV infection (Table 2).

**Figure 4:**
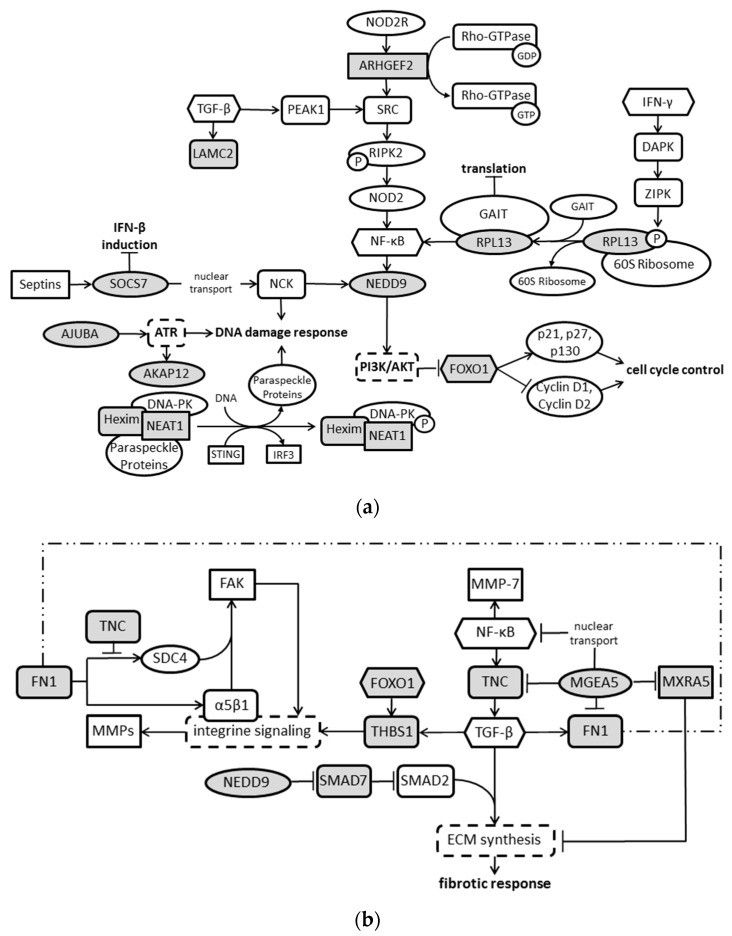
Overview of possible molecular interactions with regard to the 54 HBoV-regulated transcripts. The diagrams show the so far published interactions of HBoV-regulated target molecules, highlighted in grey, without consideration of up- or downregulation and protein–protein or protein–nucleic acid interactions. Arrows indicate induction or activation, whereas bars represent inhibitory effects. Proteins regulated by HBoV are involved in DNA damage response, translational regulation, and cell cycle control (**a**), and furthermore influence integrin signaling, which in parts overlap with TGF-β signaling, and the metabolism of the extra cellular matrix (**b**).

In addition to the proposed pathways by IPA, we independently elaborated connections and intersections of the 54 identified target molecules based on published findings. These analyses reveal that, aside from the ECM, the majority of target molecules could be allocated to DNA damage response, transcriptional/translational regulation, integrin signaling, cell cycle control (Figure 4), and calcium signaling (Table 1). As the production and exocytosis of mucins is dependent on the Ca^2+^ concentration, we evaluated the mucin production in HBoV-positive and HBoV-negative cell cultures. Compared to CuFi-1 and CuFi-5 cells, CuFi-8 showed a higher production of acid mucins in general, accompanied by an increased expression of mucins after HBoV infection in CuFi-8 cells (Figure 3b).

**Figure 5:**
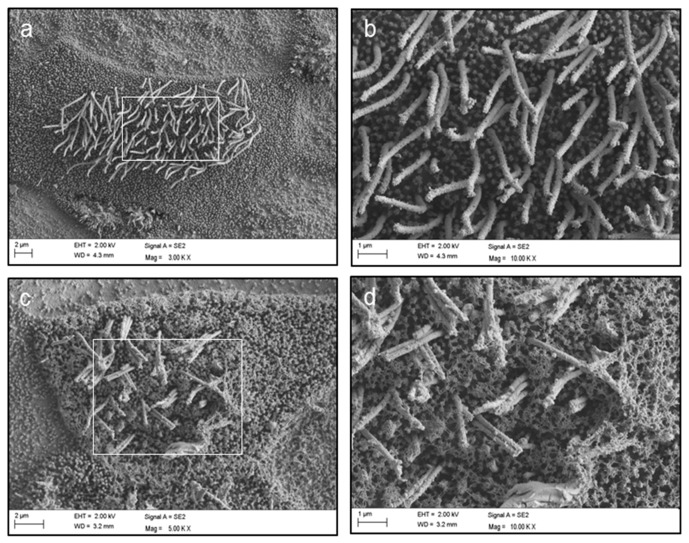
Scanning electron microscopy of mock- and HBoV-infected CuFi-8 cells. Scanning electron microscopy was used to analyze changes of the cellular surface of CuFi-8 ALIs after HBoV infection. (**a**) HBoV-negative CuFi-8 cells with a well-defined glycocalyx and functional cilia; (**b**) enlarged section of (**a**); (**c**) HBoV-positive CuFi-8 cells, which show an abnormal glycocalyx structure and destroyed cilia sticking together in bundles; (**d**) enlarged section of (**c**).

Moreover, scanning electron microscopy (SEM) indeed revealed significant changes of the cell surface regarding the cilia and the appropriate glycocalix (Figure 5).

## 3. Discussion

Aim of this study was to identify signaling pathways that are changed by the infection of HBoV1 and that may contribute to the development of solid tumors, such as non-small cell lung cancer and colorectal cancer. These cancer entities have been associated with the occurrence of HBoV1 DNA as observed earlier [2,3,4]. Moreover, our group demonstrated that profibrotic and procancerogenic cytokines/chemokines are upregulated during HBoV infection in vitro and in vivo, thus suggesting a causative role of HBoV in tumorigenesis [17]. These findings support our preliminary model, in which we have postulated that HBoV induces tumorigenesis analogous to the hepatitis B virus [16].

In the present study, we used a whole transcriptome analysis to get more detailed information of HBoV-dependent cellular changes. As expected, the HBoV affects both transcription and translation by regulating activators and inhibitors of both processes. Moreover, our results confirm the observation of Deng et al. that replication of HBoV1 may be dependent on the cellular DNA damage repair system (DDR) [19,20]. The group showed that the kinases Ataxia telangiectasia mutated (*ATM*), *ATR* (*ATM*- and *RAD3*-related) as well as *DNA-PKC* (DNA-dependent protein kinase catalytic subunit) were activated in HBoV-infected cells, which in turn is crucial for genome amplification of HBoV1, but the exact mechanism remained unknown. Our transcriptome analyses revealed that some target proteins in HBoV-infected cells are associated with *ATR*. In this context, the kinase activity of ATR is necessary for the nuclear accumulation of *NCK*, an adaptor protein of which depletion promotes apoptosis [23] and which is a target of *SOCS7*. *SOCS7* is responsible for the nuclear transport of *NCK* and also leads to an apoptotic phenotype if depleted. The fact that the *septin-SOCS7-NCK* axis intersects with the canonical DNA damage cascade downstream of *ATM/ATR* has been already known since 2007 [24] and we observed, in our analysis, elevated RNA levels of *SOCS7*, which might prevent apoptosis. The protein AJUBA is part of the DDR repression, prevents apoptosis, and controls the switch between activation of *ATR* during S phase and the general *ATR* activation after extensive DNA damage [25]. As the amount of *AJUBA* RNA was also upregulated during HBoV infection; this may further prevent HBoV-positive cells from apoptosis. These findings are compatible with the fact that HBoV does not promote apoptosis, as shown by the group from Jianming Qiu [26] and, in concert with the independent confirmation of the involvement of the DDR response in HBoV DNA replication, support the significance of the NGS transcriptome analyses presented in this study. Another factor, which is involved in the DNA damage response, is AKAP12. AKAP12 becomes phosphorylated by *ATR* and in turn is necessary for scaffolding the PKA-mediated ATR phosphorylation at S435 [27]. This potential tumor suppressor, which is downregulated in HBoV-infected CuFi-8 cells, was shown to be also reduced on mRNA levels in human lung cancers [28,29]. 

Additionally, in the presented study, deregulated RNA levels of *Hexim1* and *NEAT1* were detected. These are components of the HDP-RNP (HEXIM1-DNA-PK-paraspeckle components-ribonucleoprotein) complex that regulates DNA-mediated innate immune response [30]. The HDP-RNP complex also contains *NONO/SFPQ/PSPC1*, which dissociate upon stimulation with *ISD* (interferon stimulatory DNA) and then are available for the DDR [31,32]. Moreover, the *lncRNA NEAT1* as an essential component of paraspeckle formation seems to be dependent on the degree of cell differentiation [33]. While human embryonic stem cells (hESCs) show neither any paraspeckles nor any *NEAT1* expression, which is recovered after differentiation of the cells, one can speculate that HBoV by downregulation of *NEAT1* might restore the state of hESCs regarding regulation of some genes.

Many of the genes deregulated by HBoV are known drivers in the development of fibrosis and tumorigenesis. Among them, we identified, besides others, transcripts encoding proteins of the ECM as upregulated.

On the one hand, the ECM displays a physical network, which is necessary for mechanical stability of tissues; on the other hand, it represents a dynamic entity, which transmits signals between the intra- and extracellular space. This means that the ECM not only influences tissue morphogenesis, homeostasis and remodeling, but also has influence on gene expression and consequently to proliferation, differentiation, and apoptosis. As HBoV regulated proteins involved in the assembly of the ECM, we identified *THBS1*, *FN1*, *LAMC2* and *TNC*. All of them are known to influence cell adhesion, motility, and growth. TNC is a glycoprotein of the ECM that is regulated by the tissue microenvironment [34], of which increased expression is associated with different kinds of chronic airway inflammation, such as inflamed bronchi of smokers, interstitial pneumonia and bronchial asthma [35,36]. 

The fact that *TNC* is upregulated in HBoV-positive CuFi-8 cells further supports the hypothesis that HBoV may be involved in the development of fibrosis and tumors, as it was shown that *TNC* increases pro-collagen synthesis and activates a fibrotic response [37]. Moreover, *TNC* expression appears to be linked to lung metastasis in breast cancer [38] and leads to a downregulation of tropomyosin-1 and the *Wnt* inhibitor *Dickkopf 1*, what in turn results in the destabilization of actin stress fibers and an enhanced *Wnt* signaling [39]. Recent studies have shown that *Wnt* signaling, together with other signaling cascades, is coordinated at cilia. The fact that these pathways are often deregulated during malignant development, together with the frequent disappearance of cilia in transformed cells, suggests the possibility that deficient ciliary signaling may promote cancer [40]. *SEM* revealed defective cilia also in HBoV-infected CuFi-8 cells (Figure 5). Taking into account that *NEDD9*, which is deregulated in this study, participates in the formation and disassembly of cilia and interacts with *AurA* at the centrosome, which is necessary for cellular progression through mitosis [41,42], it seems to be possible that HBoV may indirectly lead to cellular transformation due to reprogrammed cell–matrix signaling. The assumption that HBoV triggers tumor development by inducing fibrotic alterations of the connective tissue is supported by network predictions from the IPA core analyses, which show that target proteins are involved in connective tissue organization (Figure 2b). Moreover, the translational regulator *LARP6*, which specifically regulates type I collagen expression [43], is upregulated during HBoV infection. Stabilization of collagen mRNAs and promotion of their translation lead to an increased collagen expression, which is observed in fibrotic processes.

Finally, we observed that the HBoV infection is accompanied by an elevated *CEA* expression. It is already known that HRT-cells, which are known to support the replication of the canine parvovirus, are *CEA*-positive, and both the colorectal carcinoma and non small cell lung cancer (NSCLC) frequently go ahead with an increased *CEA* production [44,45,46]. Moreover, an increased *CEA* expression was also observed in idiopathic lung fibrosis, a clinical course that could occur in HBoV-positive patients [47].

One of the markers that were upregulated with the highest statistical significance is *FN1*. In colorectal cancers, in which HBoV has been detected in about 20%, fibronectin expression is frequently upregulated and goes ahead with an upregulation of the *CEA*, which in turn is used as a diagnostic marker [48]. As *CEA* staining is a common method in pathology for the immunohistochemical characterization of tumors, we applied this method to cell cultures infected with HBoV (Figure 3a). In fact, we observed an increase of *CEA* expression associated with HBoV. In addition, increased *CEA* expression may occur in concert with increased expression of mucins [49]. Thereby, mucin expression is regulated by the Wnt/β-catenin pathway [50], the latter being significantly disturbed by the HBoV infection, as shown in this study. Thus, it was obvious to analyze to which extent the HBoV infection also influences the mucin expression. Besides, an increase in *Mucin15* RNA (ranking at place 320 of genes with altered expression within the whole transcriptome dataset of HBoV-positive CuFi-8 vs. HBoV-negative CuFi-8) was observed; the Alcian blue staining reveals an increase in mucin expression, as shown in Figure 3b, in turn supporting the hypothesis that HBoV contributes to a changed ECM, leading to a changed cell signaling.

The hypothesis that the *CEA* expression, which was also increased in our study, is associated with the HBoV infection is supported by the observation that non-HBoV-induced serious malformations in their natural hosts, as they preferably replicate in tissue with high-grade expression of CEA [45,46]. Further studies should, therefore, focus on the protein expression of the altered pathways identified in our study and should include a more differentiated kinetic analyses, which should include immediate early, early, and long-term transcription profiles to get a closer view on the putative HBoV-triggered cancerogenic mechanisms.

## 4. Materials and Methods 

### 4.1. Cells and Infections

CuFi-8 cells were grown as air–liquid interface cultures and were infected as previously described [17,18]. Infections were performed in triplicate with a recombinant HBoV wildtype generated by transfecting the plasmid pIHBoV into HEK-293 cells and subsequent harvesting [18]. Mock infections were performed by inoculating the differentiated CuFi-8 cells with exhausted HEK-293 cell culture supernatants from mock transfections (i.e., transfection with transfection reagents but w/o plasmid) that were processed exactly as the transfection media from the recombinant HBoV production [18]. Ahead of infecting CuFi-8 cells, infectious HBoV particles were harvested by collecting the medium 5 days after transfection with pIHBoV and removal of cell debris by centrifugation at 2000× *g*. This supernatant was subject to DNAse digest and subsequent qPCR for quantification of packaged genome copies. Infections were performed with MOI1. At day five, the highest HBoV replication rat was reached [18], indicated by secretion of viral particles to the basal cell culture medium and the apical surface at this time point [18]. As further mock-infected controls, CuFi-1 and CuFi-5 cells (ATCC^®^-CRL-4013^TM^ and ATCC^®^-CRL-4016^TM^) were used for excluding transcripts that were regulated unspecifically.

### 4.2. RNA Extractions and Whole Transcriptome Analyses

RNA was extracted from HBoV-infected and mock-infected CuFi-8 cells, and from CuFi-1 and -5 cells by using the RNeasy Total RNA kit (Qiagen, Hilden, Germany) according to the manufacturer’s protocol five days after infection. Whole transcriptome analyses were performed by a sequencing services vendor (MWG Eurofins, Ebersberg, Germany), according to the vendor’s protocol and strictly following the vendors’ recommendation regarding RNA quantity and quality. The approach was a HiSeq 2500 Illumina sequencing, sequencing mode 1 × 125, software HiSeq Control Software 2.2.58 (MWG Eurofins, Ebersberg, Germany), RTA 1.18.64, bclfastq-1.84 using the HiSeq SBS Kit v4. The Q30 percentage for all libraries was >96%, mapping mismatches were ≤0.5%, the read coverage mean was between 17 and 27 for all libraries, and the normalized library sizes ranged between 22,174,885.9 and 35,458,600.4. The false discovery rates of genes that were statistically significant regulated was ≤0.05 in all cases.

The resulting files reporting the transcriptional fold-change in CuFi-8 cells were analyzed using the IPA (Qiagen, Hilden, Germany) [22]. 

The IPA procedure was performed using the standard parameters recommended for core analysis which is able to identify clinical conditions, biological pathways and network interactions that display an altered regulation during the HBoV infection. Only these pathways and clinical entities identified by the core analyses were included, in which a minimum of 9 genes out of 54 specific genes (see below) displayed a statistically significantly altered regulation. The nomenclature was completely taken from the IPA platform.

### 4.3. Immunohistochemical Analyses of CuFi air–Liquid-Interface Cultures

Differentiated CuFi-ALIs were formalin-fixed paraffin-embedded according to standard procedures. Subsequently, FFPE sections were deparaffinised, hydrated and stained with the appropriate method. In case of the ECM, acid mucins were stained with the standard Alcian blue (pH 2.5) method followed by PAS. For CEA staining, the EnVision^TM^ FLEX+ detection system (Dako, Denmark) was used according to the manufacturer’s recommendations. In brief, cellular peroxidase was blocked before incubation with the primary antibody. The antibody clone CEA II-7 from Dako (Dako, Glostrup, Denmark) was used in a dilution of 1:150 (*v*/*v*). The PT-link pretreatment was performed for 20 min at 97 °C with Envision Flex Target at low pH (nitrate buffer at pH 6.1). For visualization, the respective HRP-conjugated secondary antibody and DAB chromogen, which are also included in the EnVision^TM^ FLEX+ kit, were utilized for 20 min. Mucins were visualized with the standard Alcian blue staining.

### 4.4. Scanning Electron Microscopy

SEM was used to analyze changes of the cellular surface of CuFi-8 ALIs after HBoV infection. The HAE cultures were fixed with 0.1 M cacodylate buffer containing 2.5% glutaraldehyd, 2% polyvinylpyrrolidone and 75 mM NaNO_2_ for 12 h at 4 °C. Samples were washed in 0.1 M cacodylate buffer without glutaraldehyd and subsequently incubated in a solution containing 2% arginine-HCl, glycine, sucrose and sodium glutamate for 16 h at RT. The tissue cultures were rinsed in distilled water, followed by immersion in a mixture of each 2% tannic acid and guanidine-HCl for 8 h at RT. The samples were rinsed again in distilled water and incubated in a 2% OsO_4_ solution for 8 h at RT. After three rinsing steps with distilled water the HAE cultures were dehydrated, dried in liquid CO_2_ and finally examined with a Zeiss Sigma SEM (Zeiss, Oberkochen, Germany) using 2–5 kV acceleration voltages after sputtering with gold palladium.

## 5. Conclusions

In summary, although we must concede that not all HBoV1 patients develop cancer and not all tumors are positive for HBoV, there is an increased likelihood that the virus is involved in transformation of cells and tumorigenesis and, in the susceptible host, may be an important noxious agent in cancer development. Moreover, although the used cell system is immortalized by HPV E6/E7 and hTERT genes, CuFi-8 cells remain in the sole cell culture system so far, available for studying the entire HBoV infection process from entry to release, and the results, in concert with clinical observation, justify future projects to decipher the role of HBoV in cancer development.

## Figures and Tables

**Table 1:** Overview of the 54 HBoV-specific regulated genes. The table shows that during HBoV infection, 22 transcripts are upregulated (Roman numerals), whereas 32 transcripts are downregulated (Arabic numerals).

Upregulated				Downregulated					
Target RNA	Characterization	Target RNA	Characterization	Target RNA	Characterization	Target RNA	Characterization	Target RNA	Characterization
HEXIM1 (I)	transcription regulator	TAGLN (XII)	calcium binding protein	SRSF5 (1)	splicing factor	PHLDB2 (12)	microtubule-anchoring factor	ADGRF4 (23)	G protein-coupled receptor
FN1 (II)	ECM glycoprotein	IFITM1 (XIII)	transmembrane protein	NEAT1 (2)	lnc RNA	PLA2G4F (13)	Phospholipase A	LINC0113 8 (24)	lncRNA
RHCE/RHD (III)	membrane protein	SOCS7 (XIV)	SSI protein nucleocytoplasmic shuttling protein	ERBB4 (3)	receptor tyrosine kinase	NEDD4L (14)	ubiquitin ligase	SNORD80 (25)	C/D box snoRNA
AJUBA (IV)	transcription regulator	ITPKB (XV)	kinase	AKAP12 (4)	scaffold protein	ZNF587B (15)	transcriptional inhibitor	LINC00365 (26)	lncRNA
THBS1 (V)	ECM glycoprotein	CNNM4 (XVI)	metal cation transport mediator	SNHG3 (5)	lncRNA	LINC01451 (16)	lncRNA	ANO9 (27)	membrane channel
HSPA1A/HSPA1B (VI)	chaperon	LAMC2 (XVII)	ECM glycoprotein	S100A3 (6)	Calcium binding protein	TNNI2 (17)	inhibitory subunit of the troponin complex	NFKBIZ (28)	transcription regulator
ARHGEF2 (VII)	guanine nucleotide exchange factor	CLEC7A (XVIII)	membrane receptor	NEDD9 (7)	scaffold protein	PLA2G6 (18)	phospholipase A	MIR5047 (29)	miRNA
ANKLE1 (VIII)	endonuclease	LARP6 (XIX)	translational regulator	LOC105374476 (8)	uncharacterized, affiliated with ncRNA class	THUMPD3-AS1 (19)	lncRNA	MXRA5 (30)	matrix-remodelling associated protein
SERPING1 (IX)	protease inhibitor	SIX2 (XX)	transcription regulator	ZC3H12A (9)	transcriptional activator	MGEA5 (20)	O-GlcNAcase & Acetyltransferase	CAPN8 (31)	cysteine peptidase
TNC (X)	ECM glycoprotein	RPL13 (XXI)	translational regulator	RIMKLB (10)	*N*-Acetylaspartyl-Glutamate Synthetase	PSG8 (21)	glycoprotein	LENG8 (32)	Member of leukocyte receptor cluster
TLDC2 (XI)	OXR1 protein	SMAD7 (XXII)	transcription regulator	FOXO1 (11)	transcription regulator	SH3D21 (22)	nuclear protein		

**Table 2:** Disease patterns predicted by IPA core analysis. Disease patterns with a statistically significant *p*-value (*p* ≤ 0.01) were taken into consideration. The analysis revealed that 40 out of the 54 identified transcripts, specifically regulated by the HBoV infection, are known to contribute to gastrointestinal cancer, whereas only 9 transcripts are associated with lung cancer. Numerals correspond to the ones in Table 1. Roman numerals indicate an upregulation, whereas Arabic numerals represent a downregulation.

Disease Pattern (Σ Involved Molecules)	Involved Molecules
Cancer in general (Σ 43)	I, II, III, IV, V, VI, VII, VIII, IX, X, XI, XII, XIII, XV, XVI, XVII, XVIII, XIX, XX, XXI, XXII, 1, 2, 3, 4, 6, 7, 9, 10, 11, 12, 13, 14, 17, 18, 20, 21, 22, 27, 28, 30, 31, 32
Lung cancer (Σ 9)	II, IV, V, X, XVIII, 3, 4, 7, 30
Gastrointestinal cancer (Σ 40)	I, II, III, IV, V, VI, VII, VIII, IX, X, XI, XII, XIII, XV, XVI, XVII, XIX, XX, XXII, 1, 2, 3, 4, 7, 9, 10, 11, 12, 13, 14, 17, 18, 20, 21, 22, 27, 28, 30, 31, 32
Head and neck cancer (Σ 19)	IV, 4, 27, VII, 3, II, 11, XV, XVII, 30, 18, 21, IX, XX, XXII, XII, V, X, 9
Neoplasia of epithelial tissue (Σ 41)	I, II, III, IV, V, VI, VII, VIII, IX, X, XI, XII, XIII, XV, XVI, XVII, XVIII, XIX, XX, XXII, 1, 3, 4, 6, 7, 9, 10, 11, 12, 13, 14, 17, 18, 20, 21, 22, 27, 28, 30, 31, 32
