# Correction: Schildgen, V., et al. Human Bocavirus Infection of Permanent Cells Differentiated to Air-Liquid Interface Cultures Activates Transcription of Pathways Involved in Tumorigenesis. *Cancers* 2018, *10*, 410

**DOI:** 10.3390/cancers13081774

**Published:** 2021-04-08

**Authors:** Verena Schildgen, Monika Pieper, Soumaya Khalfaoui, Wolfgang H. Arnold, Oliver Schildgen

**Affiliations:** 1 Kliniken der Stadt Köln gGmbH, Institut für Pathologie, Kliniken der Privaten Universität Witten/Herdecke mit Sitz in Köln, Ostmerheimer Str. 200, D-51109 Köln/Cologne, Germany; pieperm@kliniken-koeln.de (M.P.); khalfouis@kliniken-koeln.de (S.K.); 2 Universität Witten/Herdecke, Lehrstuhl für Biologische und Materialkundliche Grundlagen der Zahnmedizin, D-58448 Witten, Germany; wolfgang.arnold@uni-wh.de

The authors wish to make the following correction to this paper [1]: In the published version, Figure 3b appeared as a duplication of Figure 3a, while the figure legend correctly described Figure 3b with different content.

The original version of Figure 3 that has to be corrected is: 

**Figure 3:**
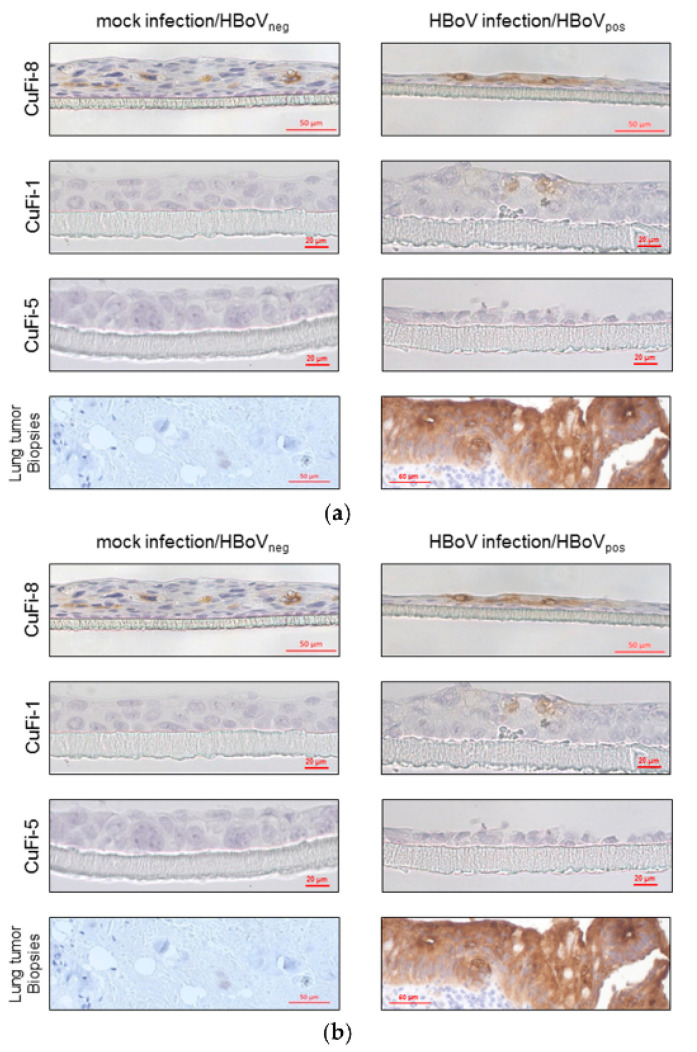
Immunohistochemical staining of CuFi-8 air–liquid-interface cultures. (**a**) Scheme 8. cells and HBoV-positive lung tumor biopsies, whereas mock-infected CuFi-8 cultures, as well as HBoV-negative lung tumors, were CEA-negative. CuFi-1 and Cufi-5 cells were not CEA-positive at all. (**b**) PAS–Alcian blue staining reveals higher production of acid mucins in CuFi-8 cells compared to those in CuFi-1 and CuFi-5 cells in general. Beyond that, there is an increased expression of acid mucins after HBoV infection in CuFi-8 cells.

It should be replaced with the following Figure 3:

We stress that these errors were purely due to human error and oversight; all corrections done do not affect or change the written proportion of the figure legend, interpretation of the results, or the final conclusions of this manuscript. The manuscript will be updated. The authors would like to apologize for any inconvenience caused. All changes have been reviewed and verified by the Academic Editors.

## Figures and Tables

**Figure 3:**
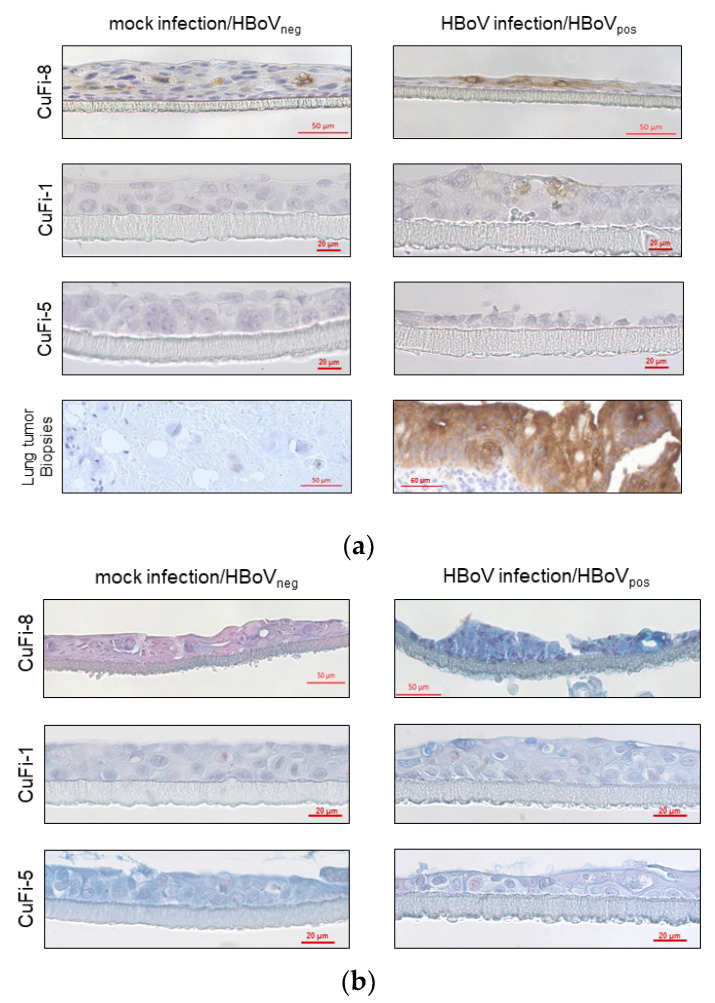
Immunohistochemical staining of CuFi-8 air–liquid-interface cultures. (**a**) Scheme 8. cells and HBoV-positive lung tumor biopsies, whereas mock-infected CuFi-8 cultures, as well as HBoV-negative lung tumors, were CEA-negative. CuFi-1 and Cufi-5 cells were not CEA-positive at all. (**b**) PAS–Alcian blue staining reveals higher production of acid mucins in CuFi-8 cells compared to those in CuFi-1 and CuFi-5 cells in general. Beyond that, there is an increased expression of acid mucins after HBoV infection in CuFi-8 cells.
